# The Battle to Reduce Maternal Deaths in Southern Lima

**DOI:** 10.1371/journal.pmed.0030016

**Published:** 2006-01-17

**Authors:** Luis Vega

## Abstract

Vega discusses the many obstacles to improving maternal health and the steps being taken to overcome them.

The Maria Auxiliadora Hospital (HAMA), a 20-year-old government hospital, serves about 2 million people living in Southern Lima, the poorest area of the city and one that is experiencing an explosive growth in population. We take care of extremely poor, malnourished patients, who have had little education and who have poor access to adequate utilities (two-thirds of homes have no safe water and no sewer).

The rapid population growth in Southern Lima began in the 1970s. People migrated to the city from the Andes, both to flee terrorism and to participate in the city's industrial development. They mostly settled in a desert area with few sources of water. Although 90% of Southern Lima is now urban, we still have a few small villages in the mountains, where people are very poor and make a living from cattle breeding and agriculture.

Working under difficult conditions with insufficient resources, and facing many other obstacles to improving maternal health (Box 1), our obstetric and gynecology team has worked hard to reduce the maternal mortality rate. In this article, I discuss the resources at our disposal, the challenges we are facing, and the steps we have taken toward reaching our goal.

**Box 1. Barriers to Improving Maternal Health in Southern Lima**

- Large number of patients and overwhelming workload
- Insufficient economic and human resources
- Bureaucracy and inefficiency in the state health system that create barriers to hiring new staff and buying new medicines and equipment—the Ministry of Health is responsible for providing health care to about two-thirds of the Peruvian population, but it has no clear policies on service delivery and long-term planning.
- Frequent changes in national government cause frequent changes in health policy. For example, the government of the 1990s supported the family-planning program, but with a new government administration in 2000, this support has been scanty and irregular, reflecting the new administration's religious, conservative orientation.
- International assistance and donations sometimes do not correlate with our priorities and more urgent needs, and they may be culturally inappropriate.
- Poor social status of women.
- A mismatch between how doctors are trained (according to a curriculum more suited to a developed country) and the actual training that would be appropriate for addressing Peru's health problems (such as better training in family planning, infectious diseases, environmental health, child health, and women's health).
- The illiteracy rate is 8.5% in the general population and 13.5% in women.
- Only 4.8% of the national budget is invested in health.

## Our Resources

HAMA has a total of 300 beds, 86 of them for obstetrics and gynecology. We have the infrastructure and technical expertise to perform highly specialized procedures, such as surgery for pelvic cancer and laparoscopic and fertility surgery. Our obstetrics and gynecology staff is composed of 26 gynecologists and obstetricians, 12 residents, 40 midwives, and 15 nurses. Box 2 gives a detailed picture of the department.

**Box 2. The Obstetrics and Gynecology Department at HAMA**

The department has three services: gynecology, obstetrics, and reproduction, each with their respective chiefs. There are eleven offices to evaluate ambulatory patients, and the appointments are scheduled from Monday to Saturday from 8 a.m. to 8 p.m. Three offices are for gynecology patients, two for low-risk obstetrics, and one each for high-risk obstetrics, gyneco-oncology, teenagers, evaluation of puerperal patients, family planning, and ultrasound studies. These offices are run by specialized doctors and residents-in-training.

We also have an emergency room, attended by two specialists who do 12-h duty shifts. In the delivery room there are two specialists and one resident from 8 a.m. to 8 p.m.; they are in charge of attending complicated deliveries, scheduled and emergency Caesarean sections, ambulatory treatment of uncomplicated abortions, and male and female sterilization. After 8 p.m. until 8 a.m., the emergency and delivery rooms are run by two specialists and two residents. Additionally, three days a week, from 8 a.m. to 8 p.m., we perform elective gynecological surgery.

Midwives are in charge of attending uncomplicated deliveries, identification of newborns, evaluation of women in normal puerperium, and counseling on family planning in the delivery room. They also are in charge of educating patients on psycho-prophylaxis, prevention of sexually transmitted infections, and promotion of cervical- and breast-cancer screening. They feed data into our computerized information system. Our nurses are in charge of the hospitalized patients and the operating theatres.

In the delivery area, there are three operating rooms for Caesarean sections and ambulatory surgery. We have the support of anesthesiologists, neonatologists, and specialized neonatology nurses wherever they are required. The hospital also has a general intensive care unit with seven beds for severely ill patients. Our blood bank runs well and we always can obtain whole blood or its derivates 24 h a day.

Our department provides health care to 575,000 women of reproductive age with a fecundity rate of 2.3 children per woman and a high rate of pregnancy in adolescents.

Eighteen percent of our pregnant patients are 19 years old or younger; this percentage has been stable during the last eight years.

HAMA is the head of the maternal and perinatal health network of Southern Lima. This network includes 15 maternal hospitals with staff composed of general physicians and midwives. These small hospitals are in charge of low-risk pregnancies and deliveries. Additionally, there is a 70-bed general hospital (Rezola Hospital) with 20 beds for gynecology and obstetrics patients located 150 km south of HAMA, which can carry out Caesarean sections. Health centers in our network communicate with each other by phone or radio; every small hospital has an ambulance to transfer patients with complications to HAMA.

In 2003 our network assisted 21,090 deliveries; 8,539 of these took place at HAMA (Figure 1), where 70% of patients have regular prenatal care (60% of pregnant women begin prenatal care before the 20th week of pregnancy). As shown in Figure 1, the number of deliveries in HAMA has increased progressively as a result of free maternal and pediatric medical care to anyone who lives in the hospital's catchment area. The proportion of deliveries at HAMA attended by different professionals is shown in Figure 2.

**Figure 1:**
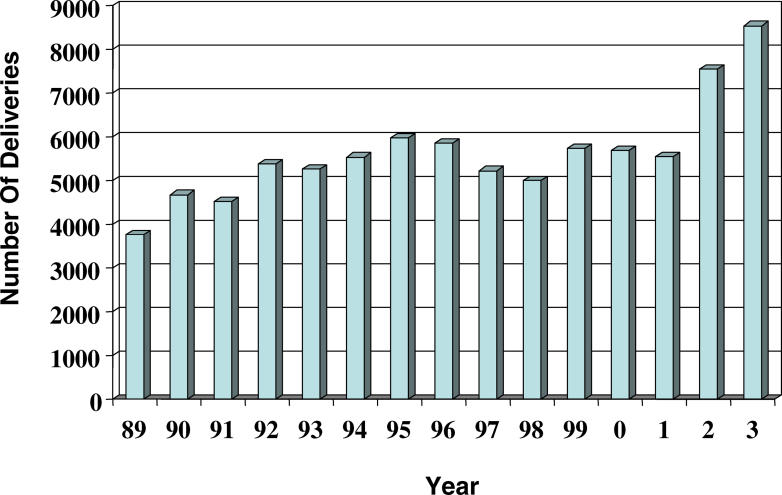
The Rising Number of Deliveries at HAMA

**Figure 2:**
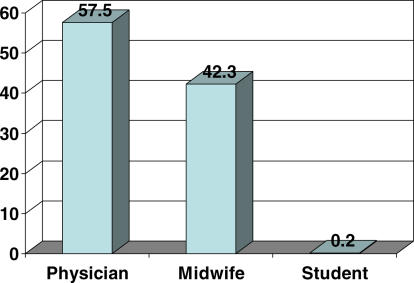
The Percentage of Deliveries Attended by a Physician, Midwife, or Student

Although the number of deliveries at HAMA has increased, there has been no increase in the hospital budget or number of health workers or any expansion of the infrastructure. Overcrowding of beds is a major problem.

## The Health Challenges

### A high maternal mortality rate

The maternal death rate at HAMA has decreased gradually from 330 per 100,000 deliveries in the late 1980s to 64.4 per 100,000 deliveries in 2003 (the 2003 rate for the whole network was 28 per 100,000 deliveries). In comparison, the national rates were 350 per 100,000 in the 1980s, 263 per 100,000 in 1996, and 152 per 100,000 in 2003.

The rate at HAMA is higher than the average rate across the whole network because we take care of patients in the network that have complications (there are almost no maternal deaths in the other health centers of our network). We also receive patients with complications from other areas—these patients are usually very ill and often need dialysis or ventilatory support.

Between 1989 and 2003, we had 111 deaths from direct obstetric causes. The most frequent causes of death were septic abortion (37 deaths), pre-eclampsia and eclampsia (35 deaths), puerperal infections (27 deaths), and hemorrhage (12 deaths, related to postpartum uterine relaxation, ruptured uterus, placental abruption, and ectopic pregnancy).

Pre-eclampsia and eclampsia complicate 14.5 % of deliveries, and one in ten of these develop HELLP syndrome (hemolysis, elevated liver enzymes, and low platelet count syndrome). Premature rupture of the membranes is the most important risk factor for puerperal sepsis, and the most severe cases arrive at our hospital with established chorioamnionitis. Tuberculosis is the most important non-obstetric cause of maternal death.

### Abortion

Induced abortion is illegal and clandestine in Peru. Safe backstreet abortions are available, but these are expensive and most of our patients are too poor to pay for such safe procedures. They risk serious complications from the cheap, unsafe procedures, but fears of being reported to the police prevent them from seeking prompt medical attention.

In 2003 we attended 2100 abortions; 90% of these were uncomplicated abortions and 10% (212) were septic. At HAMA we manage uncomplicated spontaneous abortions of less than 12-wk gestation as an ambulatory procedure using manual intrauterine vacuum devices. We manage incomplete abortions as an ambulatory procedure using a Karman-type catheter device. We realize that most of these incomplete abortions are induced abortions (generally induced by misoprostol use), but we do not ask our patients specific questions about the circumstances of their abortion. We perform the procedure and after 2 h of rest the patients leave the hospital. We have adopted this strategy to encourage patients who have had an induced abortion to seek medical attention earlier, before complications become serious. We do not perform elective abortions.

Septic abortion has been a fast-growing national problem over the last three years as a consequence of conservative governmental population policies, which have affected the regular supply of appropriate information and contraceptive methods, and because of more complicated and confusing requirements for obtaining definitive contraception (i.e., male and female sterilization).

In the 1990s, if a woman wanted to be sterilized, she only had to sign an application form. But since 2000, she now has to fill out lots of paperwork, receive at least three sessions of counseling, and undergo a 72-h period for reflection. Additionally, if she has a partner, he also has to authorize the procedure. In emergency Caesarean sections, a large proportion of patients arrive at the hospital without having fulfilled these requirements (and so unfortunately, we miss the opportunity to offer them sterilization).

### Caesarean sections

The Caesarean section rate was 31.9% in 2003. The rate has been increasing across Peru, which is due to: (1) more health centers being able to perform Caesarean sections; (2) an increase in the number of doctors trained to perform them; (3) a reduction in the rate of postoperative complications; (4) wide use of partograms with “alert lines;” (4) an increase in the incidence of pre-eclampsia and eclampsia; and (5) a rise in law trials for obstetric malpractice cases across the country.

Our main postoperative complication is puerperal infection (endometritis). We have not had anesthetic complications in the last 15 years, but in reviewing our maternal deaths, we found that 34.4% of our maternal patients who died had had a Caesarean performed.

### Contraception

Unfortunately, there has been a decline in the proportion of hospitalized patients being discharged with adequate contraception. Before 2000, 80% of our hospitalized patients left the hospital with some contraceptive method, but in 2004 this figure was only 50.6%. This decline occurred because of the lack of support for family planning from the Ministry of Health, which has led to an irregular supply of contraceptive devices and bureaucratic barriers to obtaining them.

## Action to Reduce Mortality

I believe that the most important factor in the fall in maternal mortality rate (both at HAMA and nationally) has been governmental support, which was intense in the 1990s, with the implementation of two crucial projects aimed at lowering the death rate—Project 2000, and Ten Steps for a Safe Motherhood.

There were several components to these two initiatives that helped to improve the maternal mortality rate, including a program of national hospital recertification, as well as strengthening of those hospitals selected as training centers (including HAMA). These training centers were equipped with modern medical equipment, e-mail, and other facilities. There was increased training of health personnel in these centers, including training in health quality improvement and in information processing. The training was extended to physicians, midwives, and nurses from outside these designated training centers—these professionals came to the training centers and learned while they worked with us. The training hospitals were permanently interconnected with other maternal hospitals in the country by e-mail, making it possible to share experiences. For example, staff from the training hospitals could suggest diagnostic procedures and treatments for patients with complications from other hospitals.

The two projects also involved giving strong support to the Family Planning Program with publicity campaigns and regular supply of contraceptive devices and information distributed free of charge. (This support has declined with the arrival of a new government administration in 2000.) Sterilization procedures were also offered free of charge, and sterilization campaigns were extended across the whole country, even in small towns, with portable mini-hospitals.

Other components that have helped to contribute to the reduction in maternal deaths are: (1) ambulatory management of uncomplicated, spontaneous incomplete abortions; (2) free maternal and pediatric medical care, free medicines and transportation for patients with complications from the provinces to well-equipped health centers such as HAMA; (3) creation of networks for maternal and perinatal care; (4) creation of a portable maternal medical record, used by all the hospitals in the country through a unified patient-information system; (5) development of care protocols with the participation of personnel from the network; (6) review and discussion of each maternal death in the network, to find out the factors that contributed to the patient's death (professional and administrative personnel are invited to these meetings).

In sum, we learned to work together, to exchange experiences, to improve permanently the quality of our work, to process information and to draw conclusions from it, and to appreciate the experiences at small hospitals.

## The Challenges Ahead

Despite the strides we have made against the high maternal death rates, there are many reasons why we cannot afford to be complacent. The Ministry of Health does not have long-term plans for maternal health. Short- and long-term plans—for maternal health as well as for family planning—are politically influenced, and they could change when the government changes. For example, the new government administration elected in 2000 gave little support to family planning; fortunately, in recent months it has changed its stance and has made some important changes, including distribution of the morning-after pill free of charge via every health center in the country. The national health budget has been decreasing, and many hospitals have not received new equipment. And the day-to-day reality for clinicians is that we continue to be overwhelmed by the large number of patients and the volume of work.

## Abbreviation

HAMA: Maria Auxiliadora Hospital

